# Diagnostic value of contrast-enhanced ultrasound in intravenous leiomyomatosis: a single-center experiences

**DOI:** 10.3389/fonc.2022.963675

**Published:** 2022-08-11

**Authors:** Zhitong Ge, Yahong Wang, Ying Wang, Song Fang, Hongyan Wang, Jianchu Li

**Affiliations:** Department of Ultrasound, State Key Laboratory of Complex Severe and Rare Diseases, Peking Union Medical College Hospital, Chinese Academy of Medical Sciences & Peking Union Medical College, Beijing, China

**Keywords:** intravenous leiomyomatosis, conventional ultrasound, contrast-enhanced ultrasound, gynecological tumor, ultrasonic characteristics

## Abstract

**Objective:**

Intravenous leiomyomatosis (IVL) is a rare disease, and few studies have focused on the diagnostic value of contrast-enhanced ultrasound (CEUS) in this condition. This study aimed to investigate the diagnostic value of CEUS in IVL and summarize the specific CEUS characteristics of IVL.

**Materials and Method:**

From December 2016 to March 2021, 93 patients admitted to our hospital with inferior vena cava (IVC) occupying lesions were prospectively enrolled and underwent detailed ultrasound multi-modality examinations, including conventional and contrast-enhanced ultrasound scans. The diagnostic value of CEUS and conventional ultrasound (CU) in IVL was compared, and the specific IVL signs were summarized.

**Results:**

Among the 93 patients with inferior vena cava mass, 67 were IVL while 26 were non-IVL. The inter-observer agreement of the two senior doctors was good, with Kappa coefficient = 0.71 (95% CI: 0.572–0.885). The area under the ROC curve of CU for IVL diagnosis was 0.652 (95% CI: 0.528–0.776), and its sensitivity, specificity, accuracy, positive predictive value, negative predictive value, missed diagnosis rate, and misdiagnosis rate were 61.1%, 69.2%, 63.4%, 83.7%, 40.9%, 38.8%, and 30.8%, respectively. The area under curve (AUC) for IVL diagnosis by CEUS was 0.807 (95% CI: 0.701–0.911), and the sensitivity, specificity, accuracy, positive predictive value, negative predictive value, missed diagnosis rate, and misdiagnosis rate were 82.0%, 84.6%, 82.8%, 93.2%, 64.7%, 15.4%, and 17.9%, respectively. In CEUS mode, “sieve hole sign” and “multi-track sign” were detected in 57 lesions, and the detected rate was higher than that of CU (*https://loop.frontiersin.org/people/1014187* < 0.01).

**Conclusion:**

CEUS can better show the fine blood flow inside the IVL, which is important for IVL differential diagnosis. Moreover, CEUS can obtain more information about IVL diagnosis than CU, compensating for the shortcomings of CU in detecting more blood flow within the lesion. Thus, this technique has great significance for IVL diagnosis.

## Introduction

Intravenous leiomyomatosis (IVL) is a gynecological-related tumor (1), which can invade and extend in the blood vessel and eventually involve the cardiac cavity or even the pulmonary artery, with a high risk of sudden death and pulmonary embolism (2–4). The clinical symptoms of IVL are not typical, and patients frequently have no symptoms in the early stages of the disease, resulting in missed diagnoses and misdiagnoses (5). Due to diversity and complication of IVL, few studies have focused on its ultrasonic manifestation. Conventional ultrasound has many advantages, such as radiation-free, real-time imaging, safety, convenience, and affordability, though it still has limited sensitivity to detect blood flow, especially in tiny blood vessels with low velocity (6, 7).Researchers have affirmed the feasibility of ultrasound in IVL diagnosis (8). However, conventional ultrasound often misdiagnoses IVL as a thrombus and other diseases (9–11).

CEUS is a new ultrasonic technology with a better micro blood flow imaging function that can more clearly reveal the vascular microcirculation perfusion in diseased tissue (12–14). In addition to its good safety profile and simplicity, CEUS is also patient-friendly, has no ionizing radiation, and can enable multiplanar imaging in real time (15, 16). Particularly, in patients with acute kidney disease, CEUS is a suitable imaging method since, unlike contrast-enhanced computed tomography (CE-CT) contrast agents containing iodine, ultrasound contrast agents do not cause nephrotoxicity (17). Scholars are currently using CEUS to diagnose IVL, obtaining more information than conventional ultrasound, particularly the tiny blood inside the lesion, which is critical for IVL differential diagnosis (18). IVL lesions can be well differentiated from thrombus in contrast-enhanced ultrasound (12). In addition, ultrasound plays an important role in diagnosing IVL with a better display of IVL morphology (19). However, due to a lack of a summary of specific IVL contrast-enhanced ultrasound signs, the value of contrast-enhanced ultrasound in IVL diagnosis has not been fully recognized, and there are still steps to investigate to obtain a more accurate IVL diagnosis.

Here, in this study, we aim to explore the utility of contrast-enhanced ultrasound in IVL diagnosis and summarize the specific ultrasonic signs of contrast-enhanced ultrasound in IVL.

## Material and methods

### Patients population

This study complied with the Declaration of Helsinki and the Clinical Practice Coordination Conference guidelines was approved by the Ethics Committee of Peking Union Medical College Hospital (Ethics: JS-2654). All participants signed informed consent. This study prospectively included patients treated at our hospital for IVC space-occupying from December 2016 to March 2021. A total of 93 patients with IVC space-occupying who had both conventional and contrast-enhanced ultrasounds were finally obtained, including 67 IVL and 26 non-IVL cases.

The inclusion criteria in this study were as follows: (1) patients with inferior vena cava space-occupying lesions; (2) receiving conventional ultrasonography and contrast-enhanced ultrasonography before surgery; (3) voluntary participation in this study; (4) patients undergoing surgical treatment in our hospital; (5) age ≥ 18 years. The exclusion criteria in this study were as follows: (1) incomplete preoperative clinical and imaging data; (2) unwilling to participate in this study.

### Preoperative imaging examination

#### Conventional ultrasound examination

Conventional ultrasound was performed to obtain clear ultrasound images of IVL lesions. Convex array probes C5-1MHz (IU22, EPIQ7, Philips, Netherlands) or C5-1MHz (Aplio500, i900, Canon, Japan) was used for abdominal ultrasonography. All patients received ultrasound examinations in the morning. The examination position included supine position and lateral position. A senior doctor with more than five years of vascular ultrasound experience performed the ultrasound examination. Under gray-scale ultrasound, the abdominal venous vessels, including inferior vena cava, bilateral renal veins, and bilateral iliac veins, were thoroughly scanned. Adjusting the ultrasonic parameters such as gain, depth, and focus area can display the image of IVC intravascular lesions. CDFI showed the blood flow information inside and around the lesion.

#### Contrast-enhanced ultrasound

SonoVue (Bracco, Italy), produced by the Italian Bracco company, was used as the ultrasound contrast agent. First, 5 mL normal saline was used to dilute the ultrasound contrast agent microbubble powder, followed by shaking in a suspension for standby. The amount of each injection was 1.2 mL. It was injected by mass injection through the elbow vein, and then 5 mL of normal saline was injected immediately. Mechanical index (MI) under contrast conditions was less than 0.06. The patient was laid flat without a pillow, exposing his abdomen completely. Before the contrast-enhanced ultrasound examination, the patient was instructed to keep breathing and body stability after injection of ultrasound contrast agent. After identifying the lesion area of interest based on conventional ultrasound, we quickly switched to the ultrasound contrast mode and injected 1.2 mL of ultrasound contrast agent into the elbow vein. Immediately, the machine’s built-in timing device was turned on to start synchronously recording the ultrasound contrast image and video data. Continuous observation time was not less than 2 min. After each patient’s examination, a 30-min health observation was conducted to rule out the adverse reactions to ultrasound contrast agents.

### Ultrasonic image interpretation

To avoid the difference between observers, all images were visually interpreted by two doctors with more than five years’ experience in abdominal vascular ultrasound diagnosis and recorded the ultrasonic signs of the lesions, respectively. If there were disagreements, the two doctors had to discuss and determine the final observation results. The two doctors made the ultrasonic diagnosis results without knowing the clinical and pathological data of the patients. Patients’ ultrasound images and video data were read in random order. The shape, echo type, and internal echo of the lesion in IVC were observed by conventional ultrasound. The extension path, involvement range, internal echo, the internal blood flow of the lesion, and the blood flow between the lesion and the tube wall were observed. Contrast-enhanced ultrasound was employed to observe whether the lesion was enhanced, the enhancement mode, and the shape of the contrast medium between the space-occupying lesion and the tube wall. According to video and static images, two senior doctors with more than five years’ experience evaluated and diagnosed the above results. “Sieve hole sign” and “multi-track sign” were defined as follows: (1) “Sieve hole sign” and “multi-track sign” in gray-scale mode: when the lesion was transected, two-dimensional gray-scale ultrasound showed many circular like anechoic areas, and when the lesion was transected longitudinally, there were many parallel long strip anechoic areas in it, called “sieve hole sign” and “multi-track sign” in gray-scale. (2) “Sieve hole sign” and “multi-track sign” in color Doppler mode: the circular echo and banded echo of two-dimensional gray-scale ultrasound were filled with blood flow signals, so they were called “sieve hole sign” and “multi-track sign” in color Doppler mode. (3) “Sieve hole sign” and “multi-track sign” in contrast-enhanced ultrasound mode: the transverse section showed multiple quasi-circular contrast agent microbubble condensation areas, and the longitudinal section indicated multiple spaced strip contrast agent condensation areas, called “sieve hole sign” and “multi-track sign” in contrast-enhanced ultrasound mode.

### Surgical pathology and follow-up

For patients who underwent surgical treatment to remove lesions, the pathological results were confirmed by two experienced pathologists. If suspected IVC thrombosis was effectively treated with anticoagulation and the lesion shrank or disappeared during the follow-up, it was considered a thrombosis. Alternatively, the suspected IVC thrombosis was confirmed by interventional or surgical treatment.

### Statistical analysis

The data were expressed as mean ± standard deviation (Mean ± SD) for continuous variables and numbers (percentages) for categorical variables. A t-test or a nonparametric test (Wilcoxon rank test) was used for continuous variables. The Chi-square test or Fisher exact test was utilized to analyze the count data. A weighted Kappa test was deployed to assess interobserver agreement. Statistical analysis was performed using SPSS (version 25.0; SPSS Inc., Chicago, IL, USA). MedCalc, a statistical software program (version 11.0, MedCalc, Mariakerke, Belgium), was used to determine the area under the receiver operating characteristic curve (AUC). In all analyses, statistical significance was accepted at *P* < 0.05.

## Results

### Baseline characteristics of patients

Ninety-three cases of IVC space-occupying lesions were incorporated in this study, including 67 patients in IVL group and 26 in non-IVL group confirmed by pathology. There was no gender difference between the two groups. The mean age of IVL group was 46.40 ± 6.18 years, and that of non-IVL group was 49.80 ± 12.4 years (*P* = 0.191 > 0.05). The menarche ages between the two groups were 13.46 ± 1.25 years and 13.35 ± 0.94 years, respectively (*P* = 0.95 > 0.05), without statistical difference. In IVL group, 60 patients (89.6%) had a history of hysteromyomatosis, while in non-IVL group, only two patients (7.7%) had a history of hysteromyomatosis (*P* < 0.01). In terms of clinical symptoms, patients with IVL had various symptoms. The main symptoms were lower limbs edema (n = 12) and shortness of breath (n = 11). Approximately 15 (22.4%) of IVL patients were asymptomatic. The symptoms of patients in non-IVL group were relatively less, nine patients (34.6%) had abdominal pain, and eight patients (30.8%) had no symptoms (**Table 1**).

**Table 1:** Baseline characteristics of the patients.

	IVL (n=67)	Non-IVL (n=26)	*P* -value
Age (year)	46.40 ± 6.18	49.80 ± 12.4	0.19
Age of menarche (years)	13.46 ± 1.25	13.35 ± 0.94	0.95
Uterine surgery history	60 (89.6)	2 (7.7)	<<0.01
Symptoms			<0.01
Lower limb edma	12 (17.9)	6 (23.1)	
Flustered shortness of breath	11 (16.4)	0	
Abdominal mass	1 (1.5)	0	
Lumbago and back pain	8 (11.9)	2 (7.7)	
Fatigue	2 (3.0)	1 (3.8)	
Increased menstrual	4 (6.0)	0	
Ventral belly	2 (7.1)	0	
Syncope	7 (10.4)	0	
Chest tightness after activity	1 (1.5)	0	
Vaginal bleeding	3 (4.5)	0	
bellyache	0	9 (34.6)	
Asymptomatic	15 (22.4)	8 (30.8)	

### Pathological results

The pathological results of 93 patients with IVC lesions were as follows, including 67 IVL (72.0%) and 26 non-IVL (28.0%) cases, as revealed in **Table 2**.

**Table 2:** Pathological results.

pathological result	n (%)
IVL	67 (72.0)
Non-IVL	26 (28.0)
IVC thrombosis	12 (12.9)
IVC leiomyosarcoma	14 (15.1)

### Comparison between conventional ultrasound and contrast-enhanced ultrasound images of IVL

The inter-observer agreement of the two senior doctors was good, with Kappa coefficient = 0.71 (95% CI: 0.57–0.885). By comparing the ultrasound images of IVL with conventional and contrast-enhanced ultrasound, it was found that there was no difference between them in IVL lesion morphology, continuity, protruding IVC wall, and “snake head sign,” with *P* > 0.05. However, in terms of extension route and involved range, contrast-enhanced ultrasound can find the lesions missed by conventional ultrasound, but there was no significant difference between them.

For internal blood flow display, under the condition of contrast-enhanced ultrasound, micro blood flow was found in 67 cases (100%) of IVL lesions, while conventional ultrasound only showed the presence of blood flow in 32 patients (47.8%); there was a statistically significant difference between the two (*P* < 0.01).

In addition, 24 cases (23.1%) of IVL and the inferior vena cava wall showed complete circular blood flow under conventional ultrasound conditions. In comparison, 56 patients (83.6%) with IVL lesions and the IVC wall showed full circular contrast medium under contrast-enhanced ultrasound conditions. The two groups differed statistically (*P* < 0.01; **Table 3**).

**Table 3:** Comparison of conventional ultrasound and CEUS image features in IVL group.

	CU (n=67)	CEUS (n=67)	*P* -value
Shape			*P*>0.05
solid cast	47 (70.1)	47 (70.1)	
hollow tubular	20 (29.9)	20 (29.9)	
continuity			*P*>0.05
Yes	67 (100)	67 (100)	
No	0	0	
Extension pathway			0.4919
Correct	54 (80.6)	57 (85.1)	
Missed diagnosis	13 (19.4)	10 (14.9)	
Involvement			0.4919
Correct	54 (80.6)	57 (85.1)	
Missed diagnosis	13 (19.4)	10 (14.9)	
Protruding IVC wall			*P*>0.05
Yes	0	0	
No	67 (100)	67 (100)	
Internal blood flow			*P*<0.01
Yes	32 (47.8)	67 (100)	
No	35 (52.2)	0	
Annular blood flow			*P*<0.01
Yes	43 (76.9)	11 (16.4)	
No	24 (23.1)	56 (83.6)	
Multi-track sign			*P*<0.01
Yes	20 (29.9)	57 (85.1)	
No	47 (70.1)	10 (14.9)	
Sieve sign			*P*<0.01
Yes	20 (29.9)	57 (85.1)	
No	47 (70.1)	10 (14.9)	
Snake head sign			*P*>0.05
Yes	39 (58.2)	39 (58.2)	
No	28 (41.8)	28 (41.8)	

### Comparison of diagnostic value of multimodal ultrasound For IVL

The comparison of multimodal ultrasound diagnostic values for IVL is listed in **Table 4**. In this study, 93 cases of inferior vena cava space-occupying lesions were diagnosed correctly by conventional ultrasound in 41 cases (44.1%), misdiagnosed as non-IVL in eight cases (8.6%), misdiagnosed as non-IVL in 26 cases (28.0%), and successfully diagnosed as non-IVL in 18 cases (19.3%). There were 93 cases of inferior vena cava space-occupying lesions in this study. Under the mode of contrast-enhanced ultrasound, 55 cases (59.1%) were correctly diagnosed as IVL, four cases (4.3%) were misdiagnosed as IVL, 12 (12.9%) were misdiagnosed as non-IVL, and 22 (23.7%) were correctly diagnosed as non-IVL.

**Table 4:** Diagnostic efficacy of different diagnostic methods for IVL .

Method	Sensitivity	Specificity	Accuracy	Positive predictive value	Negative predictive value	Missed diagnostic value	Misdiagnostic rate
CU CEUS CE-CT	61.1% 82.0% 85.1%	69.2% 84.6% 69.2%	63.4% 82.8% 80.6%	83.7% 93.2% 87.7%	40.9% 64.7% 64.3%	38.8% 17.9% 14.9%	30.8% 15.4% 30.8%

The AUC under ROC curve of IVL diagnosed by conventional ultrasound was 0.652 (95% CI: 0.528–0.776). The sensitivity, specificity, accuracy, positive predictive value, negative predictive value, missed diagnosis rate and misdiagnosis rate were 61.1%, 69.2%, 63.4%, 83.7%, 40.9%, 38.8%, and 30.8%, respectively.

The AUC under ROC curve of IVL diagnosed by CEUS was 0.807 (95% CI: 0.701–0.911). The sensitivity, specificity, accuracy, positive predictive value, negative predictive value, missed diagnosis rate and misdiagnosis rate were 82.0%, 84.6%, 82.8%, 93.2%, 64.7%, 15.4%, and 17.9%, respectively. The AUC under ROC curve of IVL diagnosed by enhanced CT was 0.772 (95% CI: 0.655–0.888). The sensitivity, specificity, accuracy, positive predictive value, negative predictive value, missed diagnosis rate and misdiagnosis rate were 85.1%, 69.2%, 80.6%, 87.7%, 64.3%, 14.9%, and 30.8%, respectively.

The diagnostic efficacy of the three imaging methods for IVL is displayed in **Figure 5**. The AUC of the area under the curve of CU, CEUS, CE- CT is 0.652 (95% CI: 0.528–0.776), 0.807 (95% CI: 0.701–0.911) and 0.772 (95% CI: 0.655–0.888), respectively. Comparing the area under the curve, conventional ultrasound versus contrast-enhanced ultrasound, conventional ultrasound versus contrast-enhanced CT, *P* < 0.001, Z values were 3.852 and 4.550, respectively, with a statistical difference; Contrast-enhanced ultrasound versus enhanced CT, *P* = 0.3046 > 0.05, z = 1.027, no statistical difference. The diagnostic consistency of contrast-enhanced ultrasound and enhanced CT for IVL with Kappa coefficient = 0.856 (95% CI: 0.745–0.966).

### Contrast-enhanced ultrasound features of IVL

**Figure 1:**
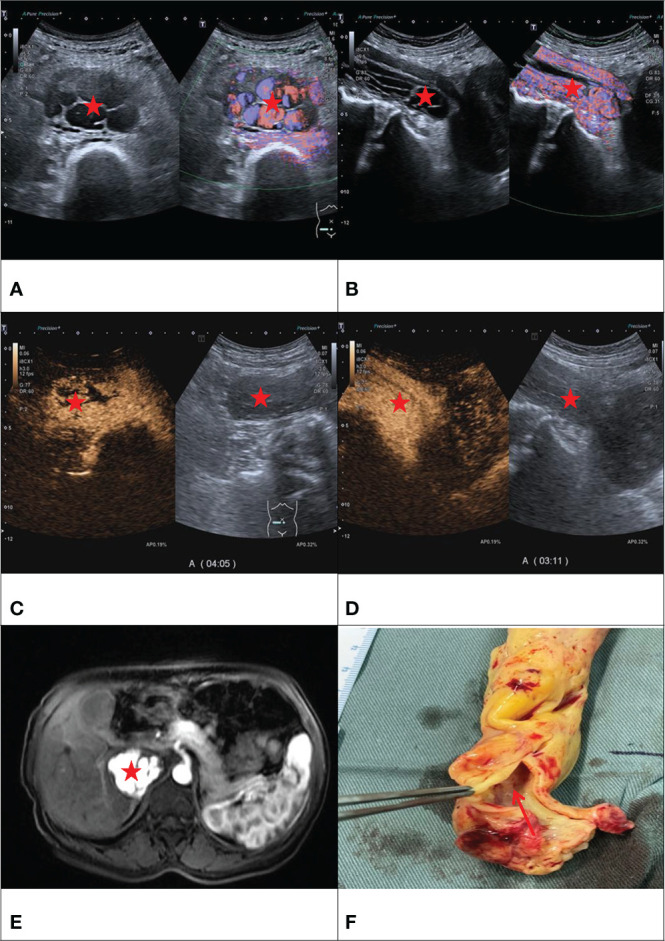
F/50Y, a physical examination found IVC occupied space, **(A)** conventional ultrasound cross-section showed sieve hole sign (Red star), **(B)** conventional ultrasound longitudinal section showed multi-track sign (Red star), **(C)** contrast-enhanced ultrasound cross-section showed sieve hole sign (Red star), **(D)** Contrast-enhanced ultrasound showed a multi-track sign on longitudinal section (Red star), **(E)** enhanced MRI cross-section showed sieve sign (Red star), **(F)** Examining the gross specimen, multiple cavities were found in it (Red arrow).

**Figure 2:**
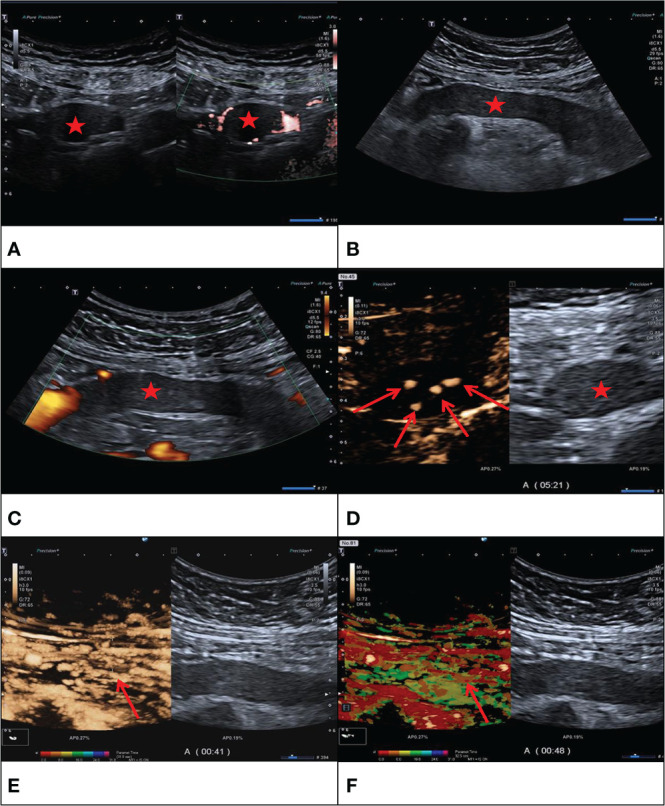
F/43Y, solid cast lesion, **(A, B)** no “sieve hole sign” and “multi-track sign” in conventional ultrasound (Red star), **(C)** no blood flow signal detected in the lesion (Red star), **(D, E)** CEUS showed the CEUS manifestations of “sieve hole sign” and “multi-track sign” respectively (Red arrow). **(F)** Contrast-enhanced ultrasound parametric imaging shows the presence of multi-track sign (Red arrow). The pathological diagnosis is IVL in this case.

**Figure 3:**
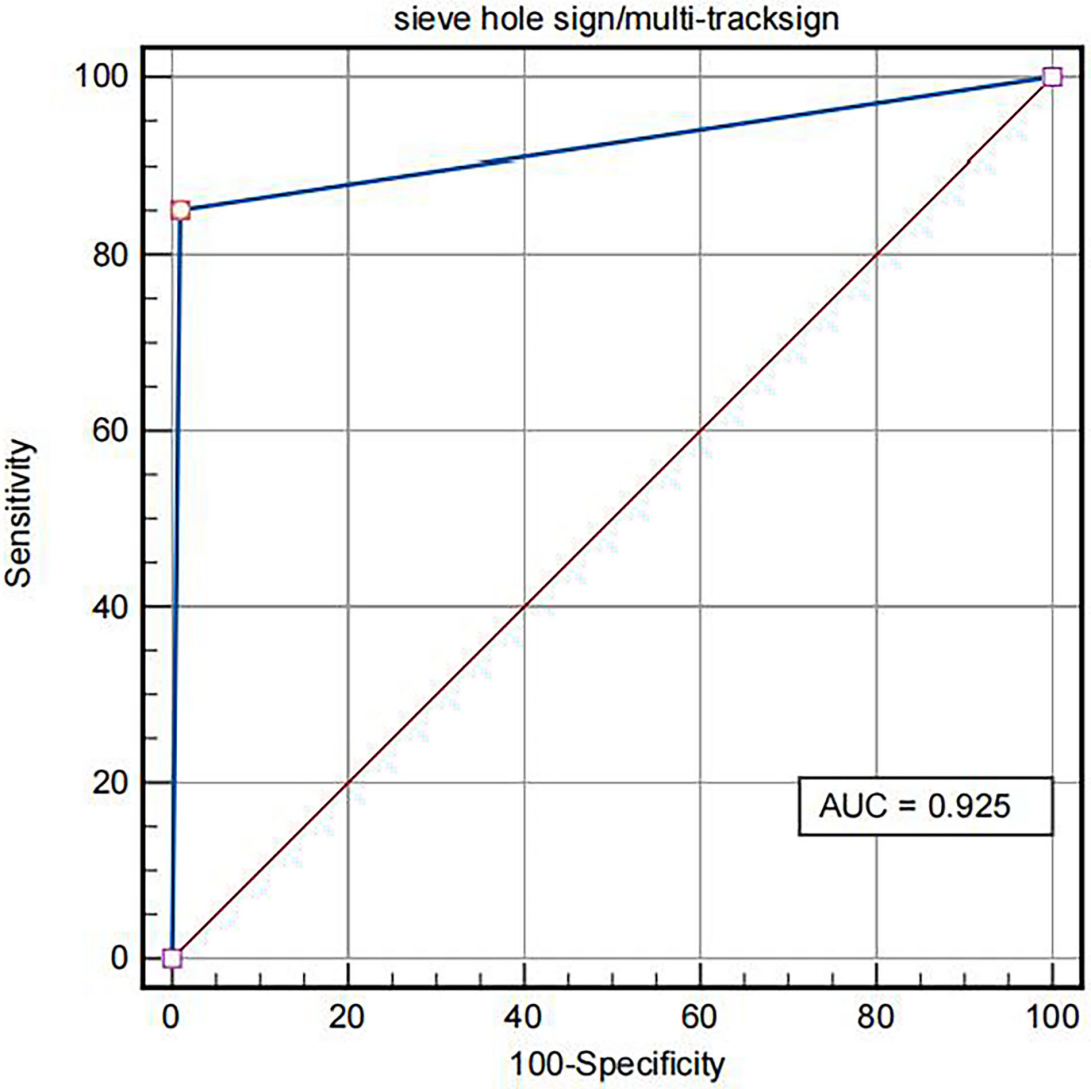
ROC of IVL diagnosed by “sieve hole sign”/”multi-track sign” in contrast-enhanced ultrasound mode.

**Figure 4:**
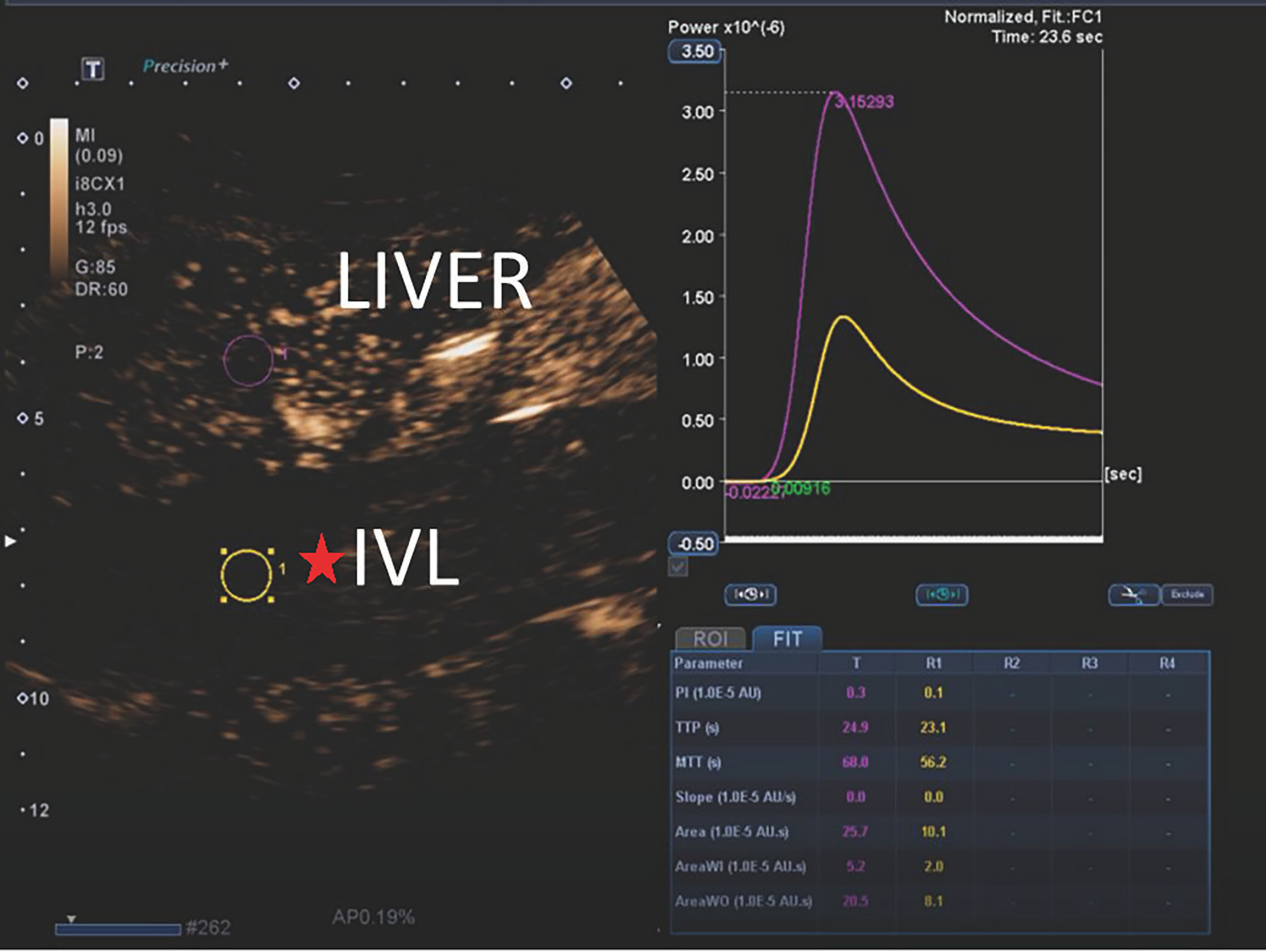
The typical CEUS curves of IVL. The peak intensity (PI), area under the curve (AUC), mean transit time (MTT), area under the curve wash in (AUC-WI), area under the curve wash out (AUC-WO) of IVL were less than liver tissue. Time to peak (TP) and curvature (Slope) of IVL was approximately equal to liver tissue. The ratio of perfusion AUI-WI/AUC-WO to the area under the washout curve of was approximately equal to liver tissue.

The contrast-enhanced ultrasound findings of 67 IVL cases are shown in **Table 3**. All 67 lesions were filled with ultrasound contrast agent microbubbles, of which 47 (70.1%) had high uneven enhancement, and 20 (29.9%) had low, uneven enhancement. The detection rate of blood flow in the lesions was 100%. In 20 cases of hollow tubular lesions, the internal cystic area was enhanced in the early stage of contrast-enhanced ultrasound, with the enhancement degree higher than surrounding parenchymal components. The transverse section was like a sieve hole, and the longitudinal section was like a multi-track, which was the performance of the “sieve hole sign” and “multi-track sign” described in the first part of the paper in the contrast-enhanced ultrasound mode (**Figure 1**). However, in 47 solid cast lesions, contrast-enhanced ultrasound showed high uneven enhancement. In 37 of these 47 cases, some continuous linear contrast microbubbles can be observed in the longitudinal axis of the lesion at the early stage of contrast-enhanced ultrasound, showing several continuous parallel linear distributions. Several significantly enhanced circular spots were in the transverse section, with enhancement intensity higher than other parts of the lesion. The typical manifestations of the “sieve hole sign” and “multi-track sign” in contrast-enhanced ultrasound mode are presented in **Figure 2**. “ Sieve hole sign” and “multi-track sign” under contrast-enhanced ultrasound were used as predictive indicators of intravenous leiomyomatosis, AUC = 0.925 (95% CI: 0.872–0.979; **Figure 3**). The diagnostic sensitivity, specificity, accuracy, positive predictive value, negative predictive value, missed diagnosis rate, and false diagnosis rate were 85.1%, 100%, 89.2%, 100%, 72.2%, and 14.9%, respectively. In this study, further analysis of the contrast-enhanced ultrasound curve of IVL revealed that compared with liver tissue, IVL has the following typical enhancement curve characteristics (**Figure 4**).

The peak intensity (PI), area under the curve (AUC), mean transit time (MTT), area under the curve wash in (AUC-WI), and area under the curve wash out (AUC-WO) of IVL are smaller than liver tissue, while the peak time (TP) and slope are similar to the enhanced patten of liver tissue, *P* > 0.05, There was no significant difference between them. In addition, no significant difference was found between the ratio of area under curve wash in (AUC-WI) and area under curve wash out (AUC-WO).

### Comparison of contrast-enhanced ultrasound and enhanced CT for IVL imaging

Compared with contrast-enhanced CT, there was no significant difference between contrast-enhanced ultrasound and contrast-enhanced CT in the internal blood flow of IVL lesions, the peripheral circular blood flow of IVL lesions or contrast agent filling, sieve hole sign, and multi-track sign, with *P* values greater than 0.05. However, compared with contrast-enhanced CT, contrast-enhanced ultrasound could observe more circular blood flow and the presence of a “sieve hole sign” and “multi-track sign” (**Table 5**).

**Table 5:** Comparison of CEUS and CE-CT image features in IVL group.

	CEUS (n=67)	CE-CT (n=67)	*P* -value
Internal blood flow			*P*>0.05
Present	67 (100)	67 (100)	
Absent	0	0	
Annular flow/circular contrast filling			0.284
Present	11 (16.4)	19 (28.3)	
Absent	56 (83.6)	48 (71.7)	
Multi-track sign			0.492
Present	57 (85.1)	54 (80.6)	
Absent	10 (14.9)	13 (19.4)	
Sieve sign			0.492
Present	57 (85.1)	54 (80.6)	
Absent	10 (14.9)	13 (19.4)	

The AUC under ROC curve of intravenous leiomyomatosis diagnosed by contrast-enhanced ultrasound was 0.807 (95% CI: 0.701–0.911). The sensitivity, specificity, accuracy, positive predictive value, negative predictive value, missed diagnosis rate and misdiagnosis rate were 82.0%, 84.6%, 82.8%, 93.2%, 64.7%, 15.4%, and 17.9%, respectively.

In this study, the AUC of the area under ROC curve of contrast-enhanced CT in the intravenous leiomyomatosis diagnosis was 0.772 (95% CI: 0.655–0.888). The sensitivity, specificity, accuracy, positive predictive value, negative predictive value, missed diagnosis rate and misdiagnosis rate were 85.1%, 69.2%, 80.6%, 87.7%, 64.3%, 14.9%, and 30.8%, respectively.

**Figure 5:**
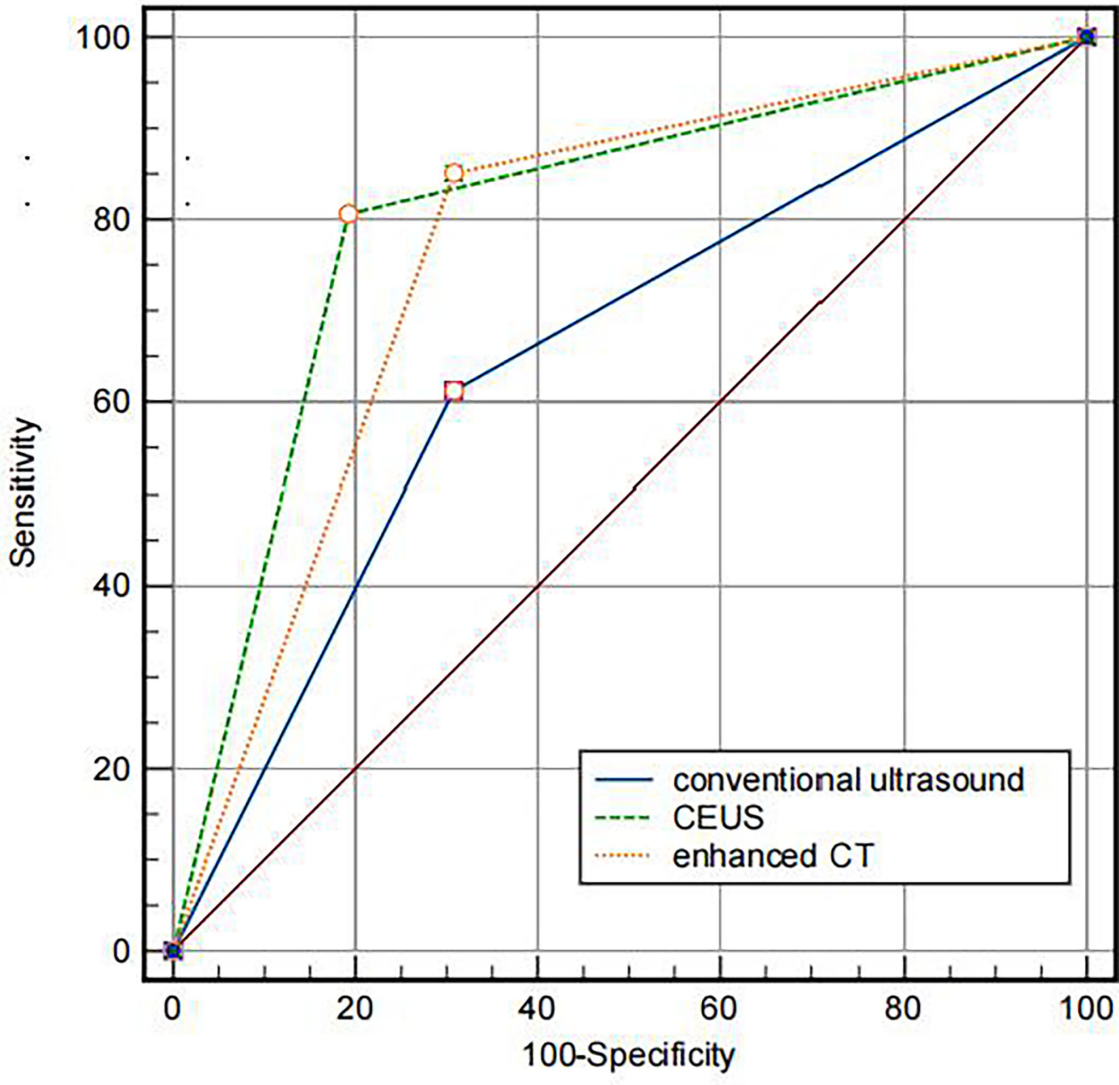
Comparison of the AUC of different imaging methods. The AUC of CU, CEUS and contrast-enhanced CT were 0.772, 0.807, 0.652, respectively.

The AUC of the area under the curve of IVL diagnosed by contrast-enhanced ultrasound and contrast-enhanced CT were 0.807 (95% CI: 0.701–0.911) and 0.772 (95% CI: 0.655–0.888), respectively. Compared with the area under the curve, contrast-enhanced ultrasound versus contrast- enhanced CT, *P* = 0.3046 > 0.05, z = 0.160, the difference was not statistically significant. Moreover, the diagnostic agreement of contrast-enhanced ultrasound and enhanced CT for IVL was good, with Kappa coefficient = 0.856 (95% CI: 0.745–0.966), indicating that contrast-enhanced ultrasound and contrast-enhanced CT have strong consistency in the diagnosis of IVL (**Figure 5**).

## Discussion

Contrast-enhanced ultrasound uses microbubble containing an inert gas, which can freely pass through capillaries and be eliminated through lung respiration. It has no nephrotoxicity and is, therefore, safer (20, 21). CEUS can reveal small blood vessels and blood perfusion that conventional ultrasound cannot. CEUS has been critical in diagnosing various clinical diseases. Ten et al. used CEUS for carotid artery examination and found that contrast-enhanced ultrasound could significantly improve the evaluation of the surface morphology of carotid plaque and the presence of an ulcer. Particularly, it can detect new blood vessels in the plaque that conventional ultrasound cannot, which is important for improving risk stratification and accurately assessing stroke risk (22). Compared to CEUS with conventional ultrasound, CEUS could more intuitively reflect the internal blood supply of intrahepatic cholangiocarcinoma lesions, thus providing more valuable information for clinical diagnosis and treatment (12, 23). In diagnosing and applying breast cancer, CEUS can fully show the micro blood flow perfusion in the lesion and more clearly show the outward invasion and growth of malignant tumors than conventional ultrasound (24). The above research is only a microcosm of the clinical application of contrast-enhanced ultrasound, which has already entirely demonstrated the superior performance of contrast-enhanced ultrasound in displaying the internal blood flow of lesions in clinical disease diagnosis. Thus, applying CEUS in IVL is significant for providing critical information for IVL diagnosis.

A recent pathological study revealed numerous reticular structures in the lesion area, and various small lumen structures were formed. The small blood vessels in the tumor are clustered and formed by a granulation-like structure under the microscope, and there is a gap between the tumor cells and vascular endothelial cells (25). Similarly, researchers also found a high expression of vascular growth factor VEGFR-3 in IVL tissue, indicating that there may be more proliferative vessels in IVL tissue (26). The pathological interpretation of the small blood vessels in IVL provided an excellent theoretical foundation for applying contrast-enhanced ultrasound in IVL.

Researchers have recently made numerous attempts to apply IVL contrast-enhanced ultrasound with satisfactory results. Ma et al. discovered that in six IVL patients, contrast-enhanced ultrasound significantly enhanced the lesions. Compared with conventional ultrasound, they can more clearly display and track the lesions range (18). The retrospective study of contrast-enhanced ultrasound images of eight patients with IVL by Luo et al. also shows that contrast-enhanced ultrasound had apparent advantages over conventional ultrasound in displaying IVL extension (12). Contrast-enhanced ultrasonography can show filling defects in intravascular and intracardiac spaces and blood supply within the lesion (27). Moreover, contrast-enhanced ultrasound can distinguish thrombus and tumor lesions well based on the enhancement of contrast agent microbubbles (28). The above studies confirmed the feasibility of contrast-enhanced ultrasound in diagnosing IVL and the display of micro blood flow in the lesion.

In this study, contrast-enhanced ultrasound and conventional ultrasound clearly showed the shape, continuity, and “snake head sign” of the lesion with no statistical difference (*P* > 0.05). Contrast-enhanced ultrasound has a higher correct display rate than conventional ultrasound in terms of the extension route and lesion involvement. However, the two were not statistically different because conventional ultrasound missed the reproductive vein lesions. If two-dimensional gray-scale ultrasound is not observed, it is also very likely to miss the diagnosis during the contrast-enhanced ultrasound, reflecting the critical significance of conventional ultrasound as the basis of other ultrasound examinations. Another reason could be that the sample size in this study is relatively small, and this difference has not been fully reflected.

In this study, 67 IVL cases showed enhanced contrast-enhanced ultrasound, indicating micro blood flow existence in IVL. However, these micro-blood flows showed significant differences in conventional ultrasound, of which only 32 cases (47.8%) were demonstrated by traditional ultrasound. The primary reason is that conventional ultrasound is insensitive to low-speed blood flow in the lesion, which is also the primary limitation of conventional ultrasound in IVL diagnosis. Failure to accurately reflect the internal blood flow of IVL lesions will make it difficult to differentiate IVL lesions from thrombosis. This study demonstrates the superior performance of contrast-enhanced ultrasound in displaying the internal blood flow of IVL lesions and can provide more valuable critical information in IVL diagnosis.

This study also found that 56 cases (85.6%) of IVL lesions showed complete circular blood flow between IVL lesions and the inferior vena cava wall by contrast-enhanced ultrasound, while this phenomenon could be observed in 24 (23.6%) patients under conventional ultrasound conditions. There was a statistical difference between the two groups (*P* < 0.01). Annular blood flow is considered an ultrasonic manifestation of no adhesion between the lesion and the wall. This study suggests that the presence of this sign has potential value in predicting IVL lesion adhesion to IVC wall in advance. The main reason for the high proportion of circular blood flow displayed by contrast-enhanced ultrasound is that it has the advantage of micro blood flow display and is not limited by blood flow direction and angle. Contrast-enhanced ultrasound technology overcomes conventional ultrasound’s technical limitations. According to literature, the IVL inferior vena cava rarely adheres to the wall (29). This study indicates that contrast-enhanced ultrasound can show this phenomenon better than conventional ultrasound.

This study found that the AUC under ROC curve of IVL diagnosed by conventional ultrasound was 0.652 (95% CI: 0.528–0.776). Its sensitivity, specificity, accuracy, positive predictive value, negative predictive value, missed diagnosis rate, and misdiagnosis rate were 61.1%, 69.2%, 63.4%, 83.7%, 40.9%, 38.8%, and 30.8%, respectively. The AUC under ROC curve of intravenous leiomyomatosis diagnosed by contrast-enhanced ultrasound was 0.807 (95% CI: 0.701–0.911). The sensitivity, specificity, accuracy, positive predictive value, negative predictive value, missed diagnosis rate and misdiagnosis rate were 82.0%, 84.6%, 82.8%, 93.2%, 64.7%, 15.4%, and 17.9%, respectively. There was a statistical difference between the two groups (*P* < 0.001), indicating that using contrast-enhanced ultrasound significantly improved the diagnostic rate of IVL, which would be useful in IVL diagnosis.

In this study, for IVL diagnosis, the area under the ROC curve of contrast-enhanced ultrasound is more significant than that of contrast-enhanced CT (ROC AUC = 0.772). The area under the curve of contrast-enhanced ultrasound is *P* = 0.3046 > 0.05, with no statistical difference, indicating that the value of contrast-enhanced ultrasound in IVL is not inferior to enhanced CT, reflecting the distinct advantages of ultrasound imaging. Moreover, contrast-enhanced ultrasound and enhanced CT have good consistency in IVL diagnosis, Kappa coefficient = 0.856 (95% CI: 0.745–0.966), which further indicates that contrast-enhanced ultrasound has good consistency in IVL diagnosis and can be used as an essential supplementary imaging examination method for IVL diagnosis besides enhanced CT.

In this study, the peak intensity (PI), area under the curve (AUC), mean transit time (MTT), area under the perfusion curve (AUC-WI), and area under the clearance curve (AUC-WO) of IVL were smaller than those of liver tissue (compared with liver), indicating that the vascular richness in IVL was smaller than the liver. The time to peak (TP) and curvature (slope) is like liver tissue, reflecting the benign histological characteristics of IVL. Furthermore, this study also found that the ratio of area under perfusion curve (AUC-WI) and area under clearance curve (AUC-WO) of IVL and liver tissue is similar, which can be used as the specific contrast enhancement curve characteristics of IVL

In this study, we found that IVL has a specific mode of ultrasound enhancement. First, in 20 cases of hollow tubular lesions, the internal cystic area was rapidly enhanced in the early stage of contrast-enhanced ultrasound, consistent with the enhancement degree and mode of the blood components in the inferior vena cava. However, the enhancement degree was higher than the peripheral parenchyma components. The transverse section was like a sieve hole, and the longitudinal section was like a multi-track, which was the performance of the “sieve hole sign” and “multi-track sign” described in the first part of the paper under the contrast-enhanced ultrasound mode. Second, in IVL of 47 solid cast lesions, ultrasound contrast agent microbubbles could be seen filling the lesions. In the early stage of ultrasound contrast, multiple continuous line-like contrast microbubbles could be seen along the longitudinal axis of lesions, showing multiple continuous parallel lines like distribution. There were multiple significantly enhanced circular spots during the transverse section, with higher enhancement intensity than lesions in other parts. Thirty-seven cases showed the “sieve hole sign” and “multi-track sign” under the typical ultrasound contrast mode. “sieve hole sign” and “multi-track sign” are the ultrasonic display of the unique lumen-like structure in IVL lesions. IVL pathological structure provided the foundation for this study’s investigation of IVL-specific ultrasonic signs. Contrast-enhanced ultrasound showed the “sieve hole sign” and “multi-track sign,” which had an excellent correlation with IVL pathological manifestations.

In conventional ultrasound, there were 20 cases (29.9%) with a “sieve hole sign” in the transverse section of lesions, while 57 cases (85.1%) with a “sieve hole sign” in contrast-enhanced ultrasound. There was a statistical difference between the two groups (*P* < 0.01). The results exhibited that contrast-enhanced ultrasound was more sensitive than conventional ultrasound in displaying the “sieve hole sign” and “multi-track sign,” particularly for solid cast IVL, which could not be distinguished from thrombus but also be used as a specific ultrasonic image for IVL diagnosis. Contrast-enhanced ultrasound showed the “sieve hole sign” and “multi-track sign” in IVL solid casting lesions, verifying the pathological basis of IVL. Meanwhile, in our study, among the 47 cases without typical “sieve hole sign” and “multi-track sign” in CU, 33 cases were correctly diagnosed as IVL by CE-CT, the rest were diagnosed as thrombus, the diagnostic accuracy rate was 70.2%. As for the 10 cases without typical “sieve hole sign” and “multi-track sign” in CEUS, they were all misdiagnosed as thrombus, the diagnostic accuracy rate was really low merely. Therefore, the diagnostic effect of CE-CT in IVL without typical “sieve hole sign” and “multi-track sign” was superior to CU, but not better than CEUS, which was probably due to the fact that CEUS could directly show the depiction of tumor vessels compared to CE-CT (30). As described in previous study, compared to CE-CT, CEUS could display more micro vessels of the lesion (31). And the diagnostic effect of CEUS and CE-CT are associated with the shape of IVL. Therefore, in this study, CEUS and CE-CT showed the same diagnostic performance in IVL with the shape of hollow tubular. However, as to IVL with solid cast, IVL could show more “sieve hole sign” and “multi-track sign” by CEUS with higher diagnostic effect, as it could show more micro vessels than CE-CT.

This study found that conventional ultrasound’s “sieve hole sign” and “multi-track sign” highly depended on lesion morphology. Only the “sieve hole sign” and “multi-track sign” appeared in the hollow tubular lesions because conventional ultrasound resolution was insufficient, and the detection of micro-blood flow in the lesions was technically restricted. Under contrast-enhanced ultrasound, the “sieve hole sign” and “multi-track sign” can achieve a perfect display of hollow tubular and solid cast IVL, independent of conventional ultrasound on lesion morphology. Thus, the “sieve hole sign” and “multi-track sign” are unified in the ultrasonic multimodal images, and the “sieve hole sign “ and “multi-track sign” under contrast-enhanced ultrasound have a broader range of applications, which will aid in the establishment and optimization of IVL ultrasonic diagnosis and treatment processes in the future.

Though CEUS has great significance in IVL diagnosis, it still has some limitation in clinical applications and the diagnostic accuracy of CEUS can be affected by objective factors such as abdominal wall fat and intestinal flatulence. Additionally, the specific signs “sieve hole sign” and “multi-track sign” in our study can apply not only to IVL occur in IVC but also in other venous system such as ovarian vein or iliac vein with IVL involvement and differentiate IVL from other IVC occupying lesions.

## Conclusion

Contrast-enhanced ultrasound has significantly higher diagnostic accuracy for IVL than conventional ultrasound. “Sieve hole sign” and “multi-track sign” can also be used as the specific signs of IVL in contrast-enhanced ultrasound diagnosis, achieving the unification of IVL-specific signs in multimodal ultrasound. Contrast-enhanced ultrasound can be an important supplement to contrast-enhanced CT in IVL diagnosis.

## Data availability statement

The original contributions presented in the study are included in the article/supplementary material. Further inquiries can be directed to the corresponding author.

## Ethics statement

The studies involving human participants were reviewed and approved by Ethics Committee of Peking Union Medical College Hospital. The patients/participants provided their written informed consent to participate in this study. Written informed consent was obtained from the individual(s) for the publication of any potentially identifiable images or data included in this article.

## Author contributions

ZG: Software, data curation, formal analysis, and visualization. ZG and YHW: Writing-original draft preparation and writing-review and editing. YW, HW, SF: Writing-review and editing. JL: Conceptualization and design, and administration and funding acquisition. All authors contributed to the article and approved the submitted version.

## Funding

This work was supported by grants from the National Natural Science Foundation of China (grant number 61971448, 82001853).

## Acknowledgments

We thank Dr. Guotao Ma, Department of Cardiac Surgery, Peking Union Medicl College Hospital, for kindly providing us with the picture shown in **Figure 1F**.

## Conflict of interest

The authors declare that the research was conducted in the absence of any commercial or financial relationships that could be construed as a potential conflict of interest.

## Publisher’s note

All claims expressed in this article are solely those of the authors and do not necessarily represent those of their affiliated organizations, or those of the publisher, the editors and the reviewers. Any product that may be evaluated in this article, or claim that may be made by its manufacturer, is not guaranteed or endorsed by the publisher.

## Abbreviations

IVL, intravenous leiomyomatosis; IVC, inferior vena cava; CU, conventional ultrasound; CEUS, contrast-enhanced ultrasound; CDFI, color Doppler flow imaging; ROC, receiver operating characteristic; AUC, area under curve; CE-CT, contrast-enhanced computed tomography; MRI, magnetic resonance imaging; MI, mechanical index; CI, confidence interval; PI, peak intensity; MTT, mean transit time; AUC-WI, area under the curve wash in; AUC-WO, area under the curve wash out; TP, time to peak.
